# Supplementary material for: Plastic Responses of Iris pumila Functional and Mechanistic Leaf Traits to Experimental Warming
Source: Plants (Basel). 2025 Mar 19;14(6):960. doi: 10.3390/plants14060960 (PMC11944494; doi:10.3390/plants14060960)

Table S1. Correlation coefficients for RGB index I_1 [134] and chlorophyll concentration (in, μgcm^{-2}) quantified by the DMSO method [135]. For each trait pair, the Pearson correlation coefficients (upper row) and the *p* values (lower row) were reported.

Trait	Chlorophyll a	Chlorophyll b	Chlorophyll a/b ratio	Total Chlorophyll
RGB index I_1	-0.6574	-0.4356	-0.6741	-0.6312
	<.0001	0.0161	<.0001	0.0002

Figure S1. Representative micrographs of *Iris pumila* leaf epidermal impressions. The images display the adaxial (A) and abaxial (B) surfaces of the same leaf. A paired *t*-test conducted on the full sample (five genotypes, with 10 micrographs per leaf side for each genotype) revealed no significant difference in stomatal density between the two surfaces ($p = 0.710$).

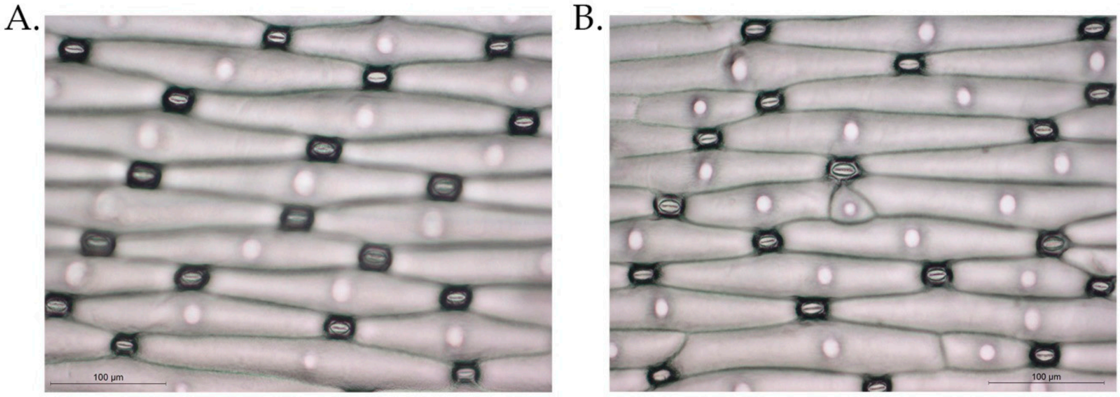

Figure S2. The relationship between RGB index I_1 [134] and chlorophyll concentration (in, μgcm^{-2}) quantified by the DMSO method [135]. The 99% prediction interval is marked with grey dashed lines.

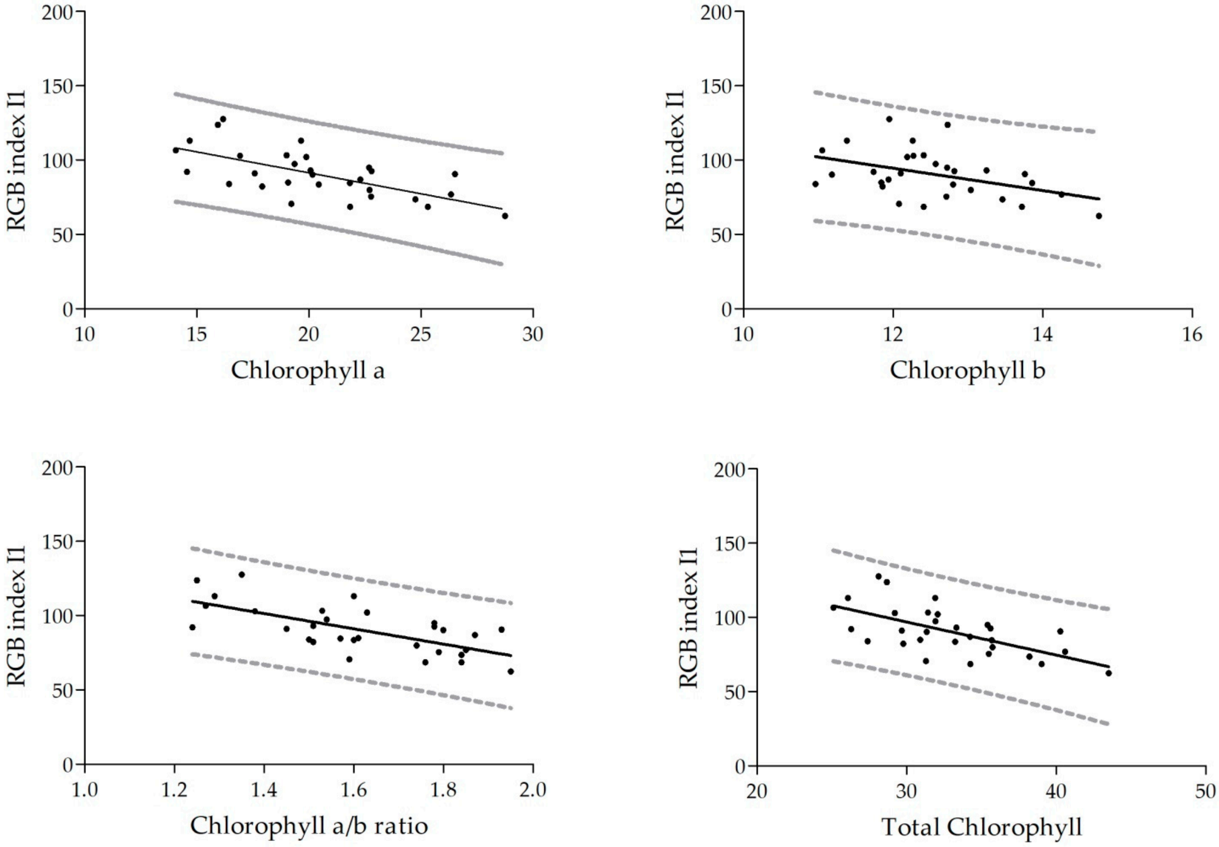

Supplement: Supplementary file 1 [file plants-14-00960-s001.zip › plants-3428304-supplementary.pdf]
